# Quantitative Proteomic Analysis of 2D and 3D Cultured Colorectal Cancer Cells: Profiling of Tankyrase Inhibitor XAV939-Induced Proteome

**DOI:** 10.1038/s41598-018-31564-6

**Published:** 2018-09-05

**Authors:** Young Eun Kim, Hyo Jin Jeon, Dahee Kim, Sun Young Lee, Ki Young Kim, Jongki Hong, Pil Jae Maeng, Kwang-Rok Kim, Dukjin Kang

**Affiliations:** 1 Center for Bioanalysis, Division of Chemical and Medical Metrology, Korea Research Institute of Standards and Science, Daejeon, 34113 Korea; 2 Therapeutic & Biotechnology Division, Korea Research Institute of Chemical Technology, Daejeon, 34114 Korea; 3 Department of Microbiology and Molecular Biology, Chungnam National University, Daejeon, 34134 Korea; 4 College of Pharmacy, Kyung Hee University, Seoul, 02447 Korea

## Abstract

Recently there has been a growing interest in three-dimensional (3D) cell culture systems for drug discovery and development. These 3D culture systems better represent the *in vivo* cellular environment compared to two-dimensional (2D) cell culture, thereby providing more physiologically reliable information on drug screening and testing. Here we present the quantitative profiling of a drug-induced proteome in 2D- and 3D-cultured colorectal cancer SW480 cells using 2D nanoflow liquid chromatography-tandem mass spectrometry (2D-nLC-MS/MS) integrated with isobaric tags for relative and absolute quantitation (iTRAQ). We identified a total of 4854 shared proteins between 2D- and 3D-cultured SW480 cells and 136/247 differentially expressed proteins (up/down-regulated in 3D compared to 2D). These up/down-regulated proteins were mainly involved in energy metabolism, cell growth, and cell-cell interactions. We also investigated the XAV939 (tankyrase inhibitor)-induced proteome to reveal factors involved in the 3D culture-selective growth inhibitory effect of XAV939 on SW480 cells. We identified novel XAV939-induced proteins, including gelsolin (a possible tumor suppressor) and lactate dehydrogenase A (a key enzyme of glycolysis), which were differentially expressed between 2D- and 3D-cultured SW480 cells. These results provide a promising informative protein dataset to determine the effect of XAV939 on the expression levels of proteins involved in SW480 cell growth.

## Introduction

Two-dimensional (2D) cell culture systems are a well-established strategy to perform *in vitro* cell-based studies. This strategy has been used extensively in cell biology research and for the discovery and development of new drugs. However, monolayer-cultured cells grown on a flat surface do not adequately represent cells *in vivo*^1^. The organizations of cells and the extracellular matrix have three-dimensional (3D) arrangements in their natural state, which means that monolayer cell cultures do not accurately mimic *in vivo* cellular environments, including cell-cell and cell-matrix communications, nutrient status, and physiological/biochemical properties^1–3^. Therefore, the cytotoxicity and activity of drugs in 2D cell culture models often do not fully match with that of tissue *in vivo*^4^. So far, diverse strategies for 3D cell culture have been developed using matrix-based or matrix-free platforms to more precisely mimic cellular microenvironments *in vivo*^5,6^. In these studies, 3D-cultured cells have shown different characteristics in cell morphology, gene/protein expression, and drug sensitivity compared to 2D-cultured cells^1,7–9^.

In proteomics, advanced tandem mass spectrometry (MS/MS) platforms coupled with on-/off-line multidimensional liquid chromatography (LC) technologies have become a fundamental way of identifying proteins and unveiling their diverse functions in various biological samples (*e.g*., sera, tissue, cells, and microbes)^10,11^. Along with diverse isotope-labeling methods (*e.g*., TMT, mTRAQ, and iTRAQ), valuable information on the quantitative alteration of targeted proteins can be obtained by measuring the relative abundance ratios of MS or MS/MS intensities of the corresponding peptide pairs labeled with light-/heavy-isotope tracers^12,13^.

Recently, various types of cells have been subjected to MS-based comparative quantitative profiling of cellular proteomes from 2D- and 3D-cultured cells, such as breast cancer, glioblastoma, and colorectal cancer (CRC) cell lines^14–16^. These studies have revealed noticeable differences in the proteomes of 2D- and 3D-cultured cells. For example, Yue *et al*. performed a stable isotope labeling of amino acid (SILAC)-based quantitative analysis of the global proteome and phosphoproteome in 2D- and 3D-cultured CRC HT29 cells^16^. These authors found that proteins related to energy metabolism were up-regulated in 3D spheroids, whereas proteins involved in cell proliferation were down-regulated.

Attempts to use 3D cell culture platforms in drug screening and testing have increased to provide reliable results in preclinical *in vitro* studies^17,18^. Compared with 2D culture models, 3D culture models tend to show resistance to anti-cancer drugs, such as melphalan, oxaliplatin, docetaxel, and paclitaxel, which has been observed in colorectal, breast, and ovarian cancer cell lines^19–21^. This difference is possibly caused by the difficulty of drug penetration into the core cells of the 3D spheroid and the increase of hypoxia-induced drug resistance^22^. In contrast, the 3D-specific anti-cancer activity of several compounds known as mitochondrial respiration inhibitors or mitotic inhibitors has been reported based on anti-cancer drug screening in 2D and 3D CRC models^23,24^. Furthermore, Adcock *et al*. showed that 3D-cultured oral cancer cells are more sensitive to anti-cancer drugs compared to 2D-cultured cells. This is due to the differences in the expression levels of the drug-target protein and the growth rate of cells between 2D and 3D culture^25^.

Wnt/β-catenin signaling is a well-known pathway that plays important roles in many biological processes, including development, differentiation, cell proliferation, and apoptosis^26,27^. Because Wnt/β-catenin signaling is aberrantly activated in various types of human cancer, especially CRC^26–28^, the inhibition of this pathway has been considered as an attractive therapeutic strategy. XAV939 is a poly-(ADP-ribosyl) transferase tankyrase inhibitor that blocks the Wnt/β-catenin signaling pathway by the regulation of axin stability^29^. The level of endogenous axin is increased upon treatment with XAV939, resulting in the down-regulation of β-catenin and its downstream signaling. Currently, the anti-cancer efficacy of XAV939 and other tankyrase inhibitors has been evaluated in preclinical *in vitro* and *in vivo* studies^30,31^. Although XAV939 is effective at blocking Wnt/β-catenin signaling in CRC cells, several studies have shown that XAV939 does not affect cell proliferation, apoptosis or cell cycle distribution of *APC-*mutant CRC cells in a conventional 2D cell culture system^32,33^. Interestingly, XAV939 efficiently suppressed the colony formation of *APC-*mutant CRC cells in a 3D culture system^29^. In addition to XAV939, other tankyrase inhibitors G244-LM (XAV939 analog) and G007-LK also showed anti-cancer effects in the colony-forming assay or xenograft models of *APC-*mutant CRC cells, despite a lack of significant effect on the viability of 2D-cultured cells^34^.

This study aimed to gain insight into the differences between 2D- and 3D-cultured SW480 cells with a focus on the XAV939-induced changes. A comparative quantitative proteomic analysis was performed on 2D- and 3D-cultured SW480 cells using isobaric tags for relative and absolute quantitation (iTRAQ) coupled with online 2D-nLC-MS/MS. We identified some significantly changed proteins when comparing 2D- and 3D-cultured SW480 cells, which were further analyzed using Gene Ontology (GO) term and Kyoto Encyclopedia of Genes and Genomes (KEGG) pathway enrichment approaches. Moreover, we explored the factors involved in the 3D-culture-selective growth inhibitory effect of XAV939 on SW480 cells. Based on the quantitative profiling of XAV939-induced proteome in 2D- and 3D-cultured SW480 cells, we identified and validated gelsolin and lactate dehydrogenase A as novel XAV939-target proteins that are differentially expressed between 2D- and 3D-cultured SW480 cells.

## Results and Discussion

### Quantitative Proteomic Analysis of 2D- and 3D-cultured SW480 Cells Treated with XAV939

**Figure 1:**
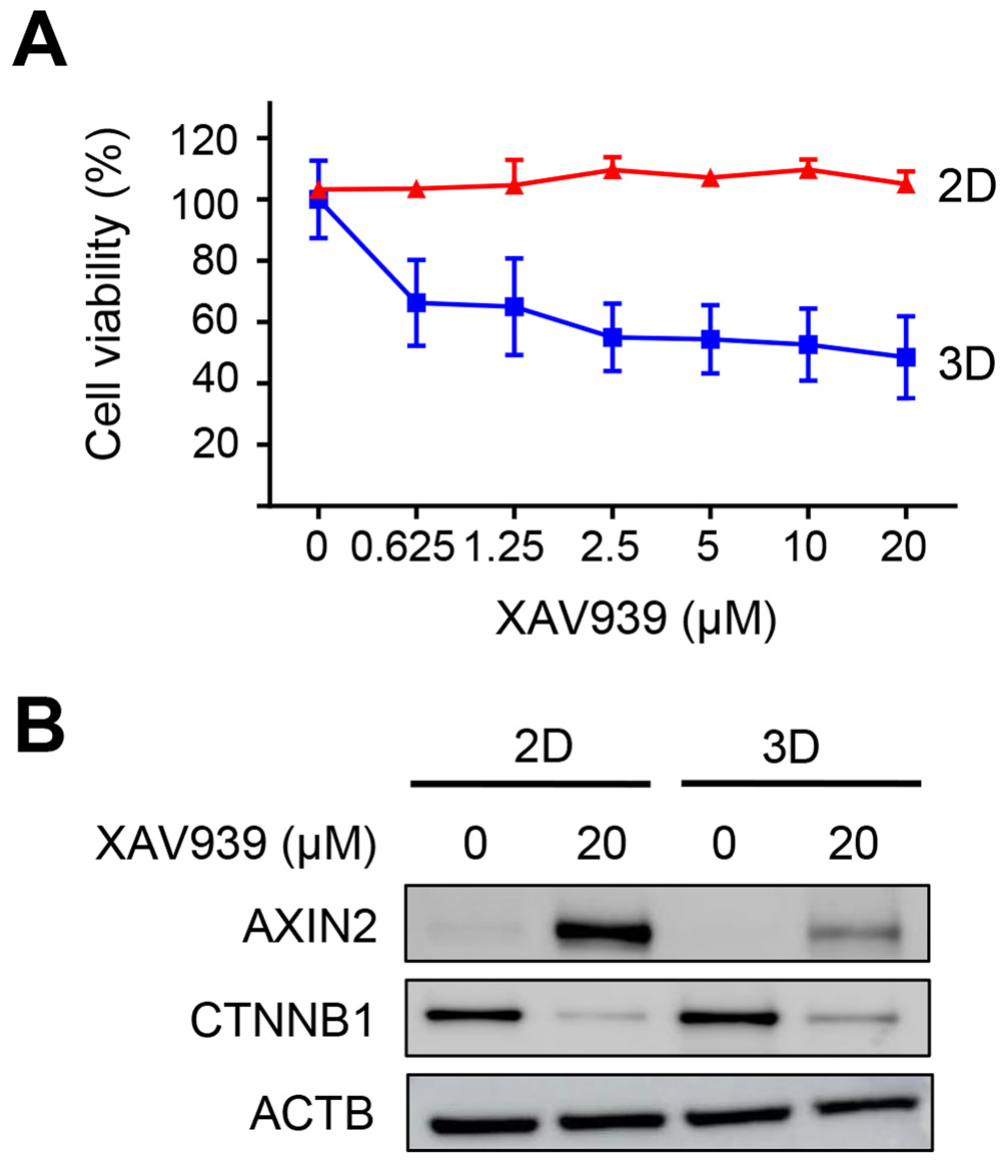
Differential Effects of XAV939 on the cell growth of 2D- and 3D-cultured SW480 cells. (**A**) Dose-dependent curves of 2D- and 3D-cultured SW480 cells treated with XAV939 at indicated concentration (0.625–20 μM). Data are shown as means ± SD from three independent experiments. (**B**) Protein expression levels of AXIN2 and CTNNB1 by western blotting in 2D- and 3D-cultured SW480 cells treated or not treated with 20 μM of XAV939. Full-length blots are presented in Supplementary Fig. S2. ACTB was used as a loading control. AXIN2, axin-2; CTNNB1, β-catenin; ACTB, β-actin.

We investigated the effects of XAV939 on the cell growth of 2D- and 3D-cultured human CRC SW480 cells. *APC-*mutant SW480 cells, which are constitutively active in the Wnt/β-catenin signaling pathway, were grown in 2D and 3D cultures and treated with various concentrations of XAV939 ranging from 0 to 20 μM. XAV939 did not show any noticeable anti-proliferation effects on 2D-cultured SW480 cells, whereas it suppressed the growth of the 3D-cultured cells in a dose-dependent manner (Fig. 1A). Compared to the untreated 3D-cultured cells, SW480 cells grown in 3D culture showed 48 ± 12% cell survival in the presence of 20 μM XAV939. However, SW480 cells were completely resistant to the same concentration of XAV939 in 2D culture.

The XAV939-mediated inhibition of tankyrase induces the stabilization of AXIN2 (axin-2, a negative regulator of Wnt/β-catenin signaling) and the reduction of CTNNB1 (β-catenin) protein levels. Therefore, we next examined the expression levels of AXIN2 and CTNNB1 in 2D- and 3D-cultured SW480 cells after treatment with XAV939. XAV939 treatment led to the up-regulation of AXIN2 and the down-regulation of CTNNB1 in both 2D- and 3D-cultured SW480 cells (Fig. 1B). The stabilization of AXIN2 by XAV939 treatment was more effective in 2D than 3D-cultured SW480 cells, but CTNNB1 was similarly down-regulated in 2D- and 3D-cultured SW480 cells. These results indicate that XAV939 effectively impairs the Wnt/β-catenin signaling in both 2D- and 3D-cultured SW480 cells.

**Figure 2:**
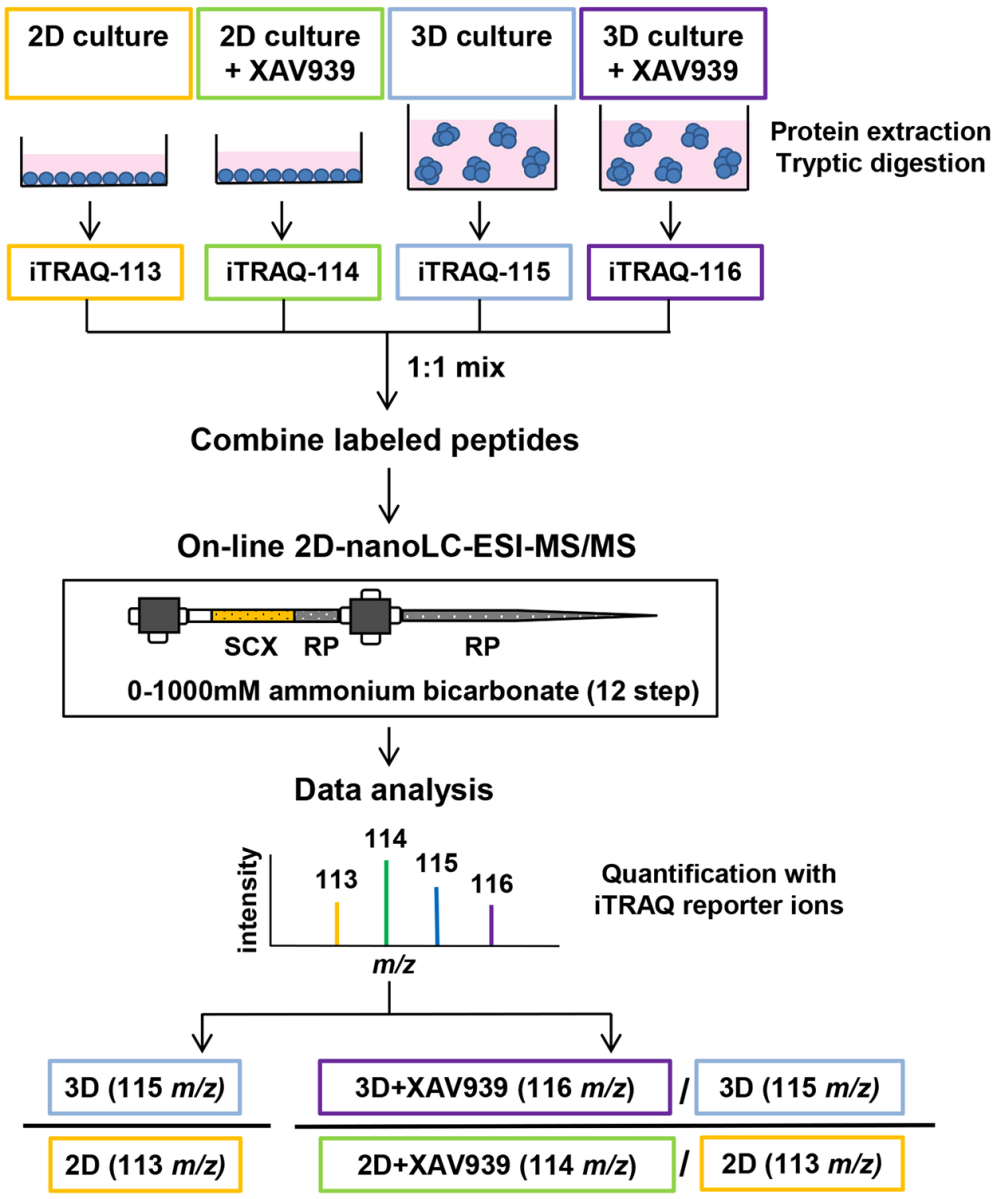
Work flow for iTRAQ-based quantitative proteomic experiment.

The quantitative proteomic analysis was carried out using iTRAQ labeling coupled with online 2D-nLC-MS/MS to gain insight into the global changes between 2D- and 3D-cultured SW480 cells and to reveal factors involved in the 3D culture-specific growth inhibitory effects of XAV939 on SW480 cells (Fig. 2). We compared the proteomes of 2D- and 3D-cultured SW480 cells treated with either 20 μM of XAV939 or DMSO as a control. Proteins extracted from these four samples were tryptically digested and labeled with different isobaric tags: 113 tag for 2D-cultured cells; 114 tag for 2D-cultured cells treated with 20 μM of XAV939; 115 tag for 3D-cultured cells; and 116 tag for 3D-cultured cells treated with 20 μM of XAV939. The labeled peptides were equally pooled, followed by online 2D-nLC-MS/MS analysis (see Materials and Methods). The quantitative analysis relied on measuring the relative intensities of iTRAQ reporter ions with different masses (*m/z* 113, 114, 115, and 116) produced during the fragmentation of precursor ions in MS/MS experiments. We calculated iTRAQ 115/113 ratios for the comparison of 2D- and 3D-cultured cells and iTRAQ 116/115 versus 114/113 ratios for the comparison of XAV939-induced proteomic changes between 2D- and 3D-cultured cells. A total of 4854 proteins were quantified with confidence corresponding to peptide and protein FDR < 0.01 and with at least two unique peptides per protein (Table S1 in Supplementary Information). Both quantitative datasets for iTRAQ ratios 115/113 and 116/115 versus 114/113 followed a normal distribution (Fig. S1 in Supplementary Information).

### Comparison of Proteomic Differences between 2D- and 3D-Cultured SW480 Cells

**Figure 3:**
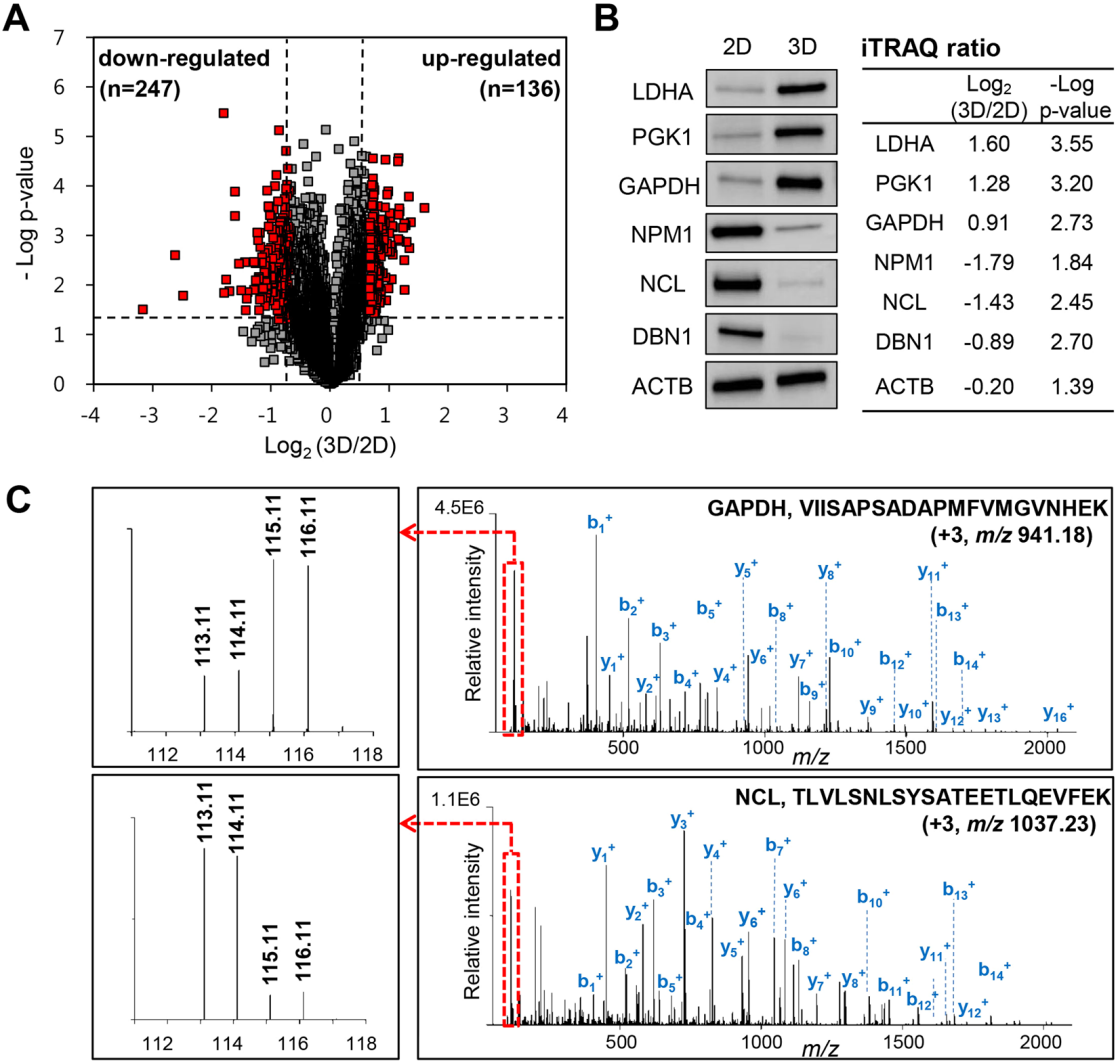
Proteomic comparison of SW480 cells between 2D and 3D culture. (**A**) Volcano plot of quantified proteins constructed from log_2_ fold change (x-axis) and –log p-value (y-axis). The threshold for determining differential expression is indicated by dashed lines (p value ≤ 0.05, fold-change > 1.6). Red dots indicate significantly up-regulated and down-regulated proteins. (**B**) Validation of differentially expressed proteins in 2D- and 3D-cultured SW480 cells using western blot analysis. Up-regulation of LDHA, PGK, and GAPDH and down-regulation of NPM, NCL, and DBN in 3D culture were observed in comparison to 2D culture. ACTB was used as a control. Full-length blots are presented in Supplementary Fig. S3. iTRAQ ratios and –log p-values of these proteins are shown in the right side of blots. LDHA, lactate dehydrogenase A; PGK1, phosphoglycerate kinase; GAPDH, glyceraldehyde 3-phosphate dehydrogenase; NPM1, nucleophosmin; NCL, nucleolin; DBN1, drebrin; ACTB, β-actin. (**C**) Representative MS/MS spectra of VIISAPSADAPMFVMGVNHEK for identified GAPDH (top) and TLVLSNLSYSATEETLQEVFEK for identified NCL (bottom). Left boxes represent spectra of iTRAQ reporter ions. 113.11, 2D culture; 114.11, 2D culture treated with XAV939; 115.11, 3D culture; 116.11, 3D culture treated with XAV939.

To compare the proteomes of 2D- and 3D-cultured SW480 cells, statistically significant differences in protein abundance were determined based on the fold change with a cut-off of 1.6 and a t-test p-value threshold of 0.05 (red dots in Fig. 3A). We identified 136 up-regulated proteins and 247 down-regulated proteins in 3D compared to 2D culture (Tables S2 and S3 in Supplementary Information). To validate the global proteomic data, the expression levels of several selected proteins were confirmed using western blot analysis. LDHA (lactate dehydrogenase A), PGK1 (phosphoglycerate kinase 1), and GAPDH (glyceraldehyde 3-phosphate dehydrogenase) were highly expressed in 3D than 2D-cultured cells, whereas the expression levels of NPM1 (nucleophosmin), NCL (nucleolin), and DBN1 (drebrin) were lower in 3D-cultured cells (Fig. 3B). These results are consistent with previous iTRAQ-based quantitative analyses. Figure 3C shows representative MS/MS spectra for tryptic peptides VIISAPSADAPMFVMGVNHEK (*m/z* 941.18 with 943 charge) and TLVLSNLSYSATEETLQEVFEK (*m/z* 1037.23 with 103 charge), which are derived from GAPDH and NCL, respectively.

**Figure 4:**
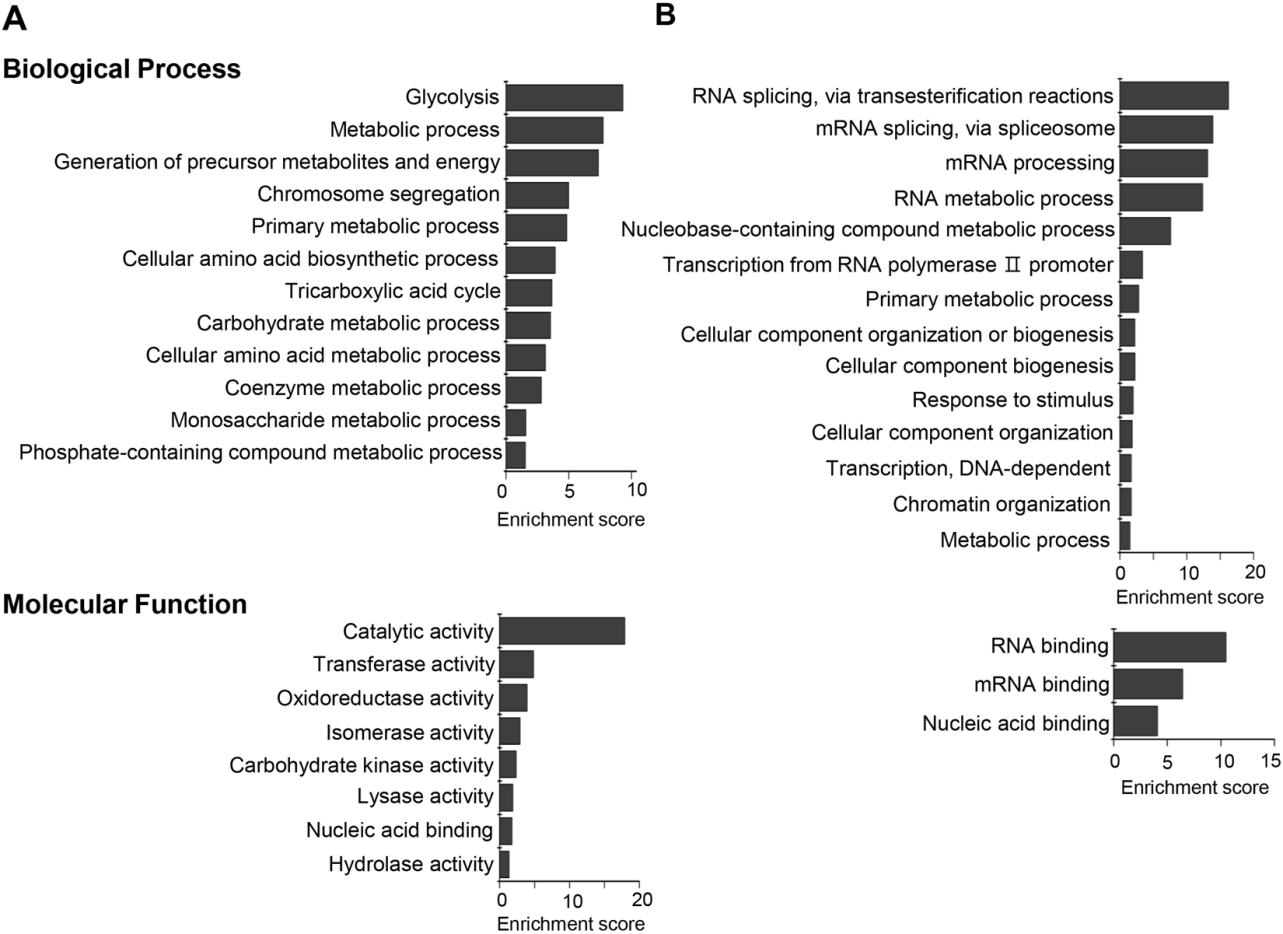
Gene ontology (GO) enrichment analysis of differently expressed proteins between 2D- and 3D-cultured SW480 cells. The up-regulated (**A**) and down-regulated (**B**) proteins were classified according to their biological processes (top) and molecular functions (bottom). The enrichment score of GO terms was calculated by –log p-value.

We also analyzed the GO and KEGG pathway enrichment to characterize functionally the significantly changed proteins between 2D- and 3D-cultured SW480 cells. The differentially expressed proteins were classified according to their biological processes and molecular functions (Fig. 4). The 12 GO terms of biological processes including glycolysis, metabolic processes, and amino acid biosynthesis were enriched in 3D-up-regulated proteins. The 14 GO terms were enriched by proteins down-regulated in 3D culture, and these proteins were involved in DNA/RNA processing and cellular component organization. For the category of molecular functions, up-regulated proteins were related to enzyme activities such as catalytic activity, transferase, and oxidoreductase activity, while DNA/RNA binding proteins were enriched in proteins down-regulated in 3D culture. The results from the KEGG pathway analysis are shown in Table 1. The 3D-up-regulated pathways included metabolic pathways and glycolysis/gluconeogenesis, whereas oxidative phosphorylation was down-regulated in 3D culture. These results indicate that 3D-cultured cells tend to depend on glycolysis rather than mitochondrial oxidative respiration for energy production, which possibly results from oxygen or nutrient gradients that occur in 3D microenvironments. In agreement with these results, up-regulation of the HIF-1 (hypoxia-inducible factor-1) signaling pathway was also observed in 3D culture. In addition to metabolic changes, the gap junction pathway including cytoskeletal proteins (α- and β-tubulin) and MAPK (mitogen-activated protein kinase) signaling proteins (MEK1 and ERK1), which contributes to intercellular communications, was down-regulated in 3D culture. The down-regulation of proteins involved in DNA/RNA processing and cellular component biogenesis/organization is correlated with the relatively slower growth of 3D-cultured cells in comparison to those in 2D-cultured cells.

**Table 1:** Significantly enriched KEGG pathways of differentially expressed proteins between 2D- and 3D-cultured SW480 cells.

Pathway ID	Pathway description	Count	FDR
**Up-regulated pathway in 3D culture**			
1100	Metabolic pathways	44	4.99E-20
1200	Carbon metabolism	18	5.40E-19
10	Glycolysis/Gluconeogenesis	12	3.53E-13
1230	Biosynthesis of amino acids	12	1.91E-12
4540	Gap junction	9	1.98E-07
30	Pentose phosphate pathway	6	6.67E-07
4145	Phagosome	8	1.33E-04
230	Purine metabolism	8	3.11E-04
4066	HIF-1 signaling pathway	4	4.89E-02
**Down-regulated pathway in 3D culture**			
3040	Spliceosome	28	1.5E-25
190	Oxidative phosphorylation	8	1.2E-02

Notes: Significant KEGG pathways identified in STRING network, Count: the number of differentially expressed proteins.

Abbreviations: KEGG, Kyoto Encyclopedia.

Our results are mostly in agreement with those reported by Yue *et al*. in that proteins involved in the metabolic pathway, amino acid biosynthesis/metabolism, HIF-1 signaling pathway, and DNA replication were significantly different between 2D and 3D cultures of CRC HT29 cells. However, the changes in abundance of proteins related to energy metabolism were not consistent with this previous report^16^. Although the glycolysis/gluconeogenesis pathway was highly enriched in the 3D-up-regulated proteins in both results, proteins associated with oxidative phosphorylation were down-regulated in this study (ATP5D, ATP5J, COX17, COX6B1, NDUFA8, NDUFAB1, UQCRB, and UQCRH), but up-regulated in the results of Yue *et al*. (COX6C, COX7A2, NDUFA13, NDUFA8, PPA2, SDHA, SDHB, SDHC, UQCR10, UQCRB, UQCRC1, etc.). He *et al*. also reported global proteomic comparisons of 2D- and 3D-cultured cells using glioma U25l cells, which also showed the enrichment of glycolysis-associated terms in 3D-up-regulated proteins^15^. This proteomic study identified seven oxidative phosphorylation-associated proteins that were down-regulated in 3D culture (AHCY, ATP5A1, ATP5F1, COX4I1, UQCRC1, UQCRC2, and PPA1). Taken together, these results suggest that metabolic reprogramming to increase glycolysis commonly occurred in 3D culture, but the regulation of mitochondrial oxidative phosphorylation (either activation or inactivation) could be influenced by the types of cells, 3D cell culture systems, spheroid sizes, or other factors.

### Comparison of XAV939-Induced Proteomic Changes between 2D- and 3D-Cultured SW480 Cells

**Figure 5:**
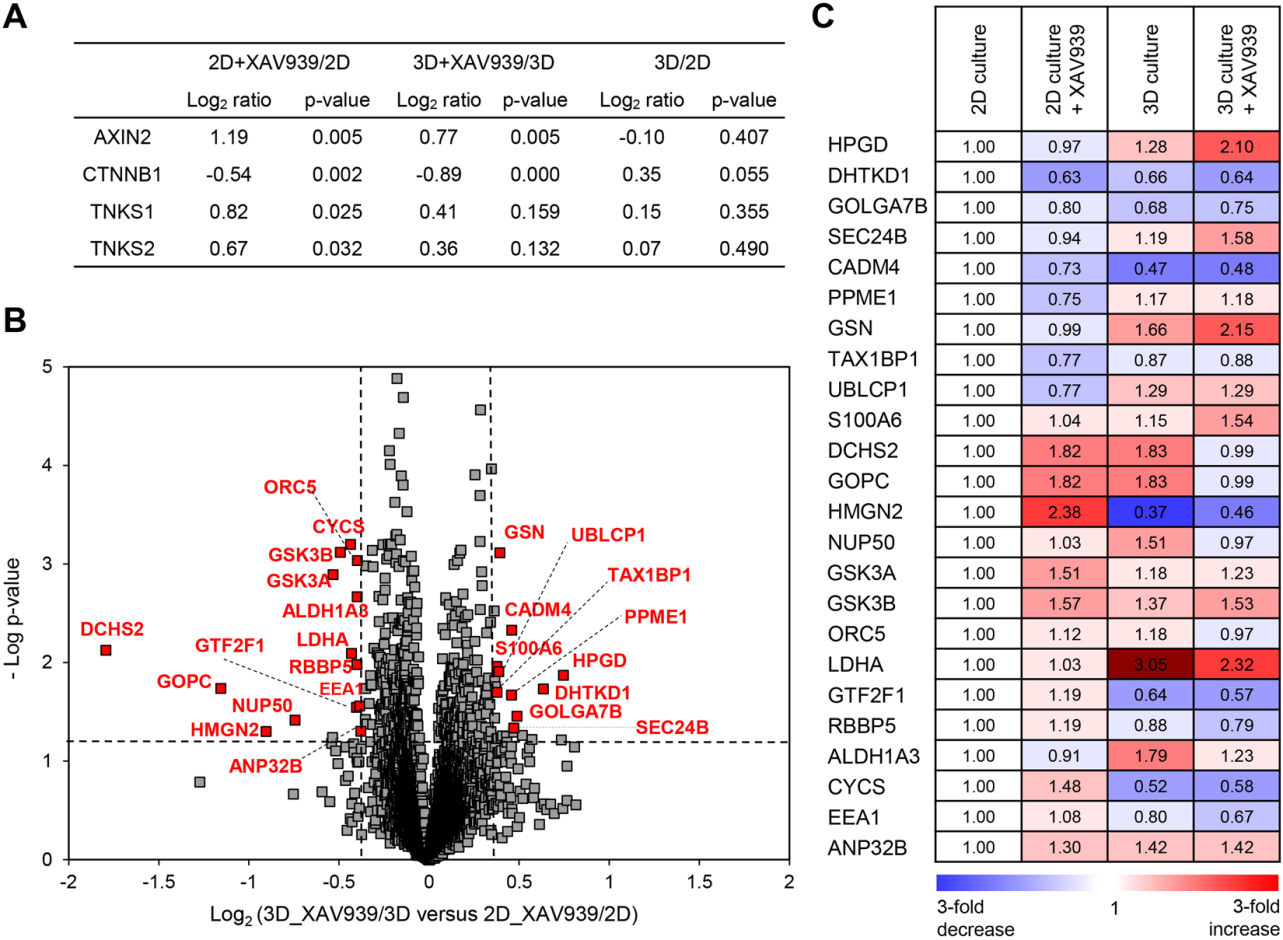
Proteomic comparison of the XAV939-induced changes in 2D- and 3D-cultured SW480 cells. (**A**) iTRAQ ratios of AXIN2, CTNNB1, TNKS1, and TNKS2 in 2D culture treated with XAV939/2D culture, 3D culture treated with XAV939/3D culture and 3D culture/2D culture. P-value was calculated with student’s t-test. AXIN2, axin-2; CTNNB1, β-catenin; TNKS1, tankyrase 1; TNKS2, tankyrase 2; ACTB, β-actin. (**B**) Volcano plot of quantified proteins for 3D culture treated with 20 μM XAV939/3D culture versus 2D culture treated with 20 μM XAV939/2D culture. The vertical dashed lines represent 1.3-fold change, and the horizontal dashed line indicates a p-value of 0.05. Significantly changed proteins are shown as red dots with gene names in the plot. (**C**) Relative protein expression levels of 24 proteins that were significantly changed between 2D- and 3D-cultured SW480 cells in response to XAV939. Values are expressed as fold changes from iTRAQ data and protein expression levels were normalized to 2D culture.

Next, we analyzed established XAV939-target proteins containing TNKS1 (tankyrase 1), TNKS2 (tankyrase 2), AXIN2, and CTNNB1. The following iTRAQ ratios of these proteins were calculated: 114/113 (2D culture treated with XAV939/2D culture), 116/115 (3D culture treated with XAV939/3D culture), and 115/113 (3D culture/2D culture). After XAV939 treatment, AXIN2, TNKS1, and TNKS2 were remarkably up-regulated in both the 2D and 3D cultures (Fig. 5A). However, the up-regulation of TNKS1 and TNKS2 was not statistically significant in 3D culture (TNKS1, p-value . 0.159; TNKS2, p-value .10.132). Although the degree of the up-regulation of AXIN2, TNKS1, and TNKS2 was slightly higher in 2D than 3D culture, CTNNB1 was successfully down-regulated in both 2D and 3D culture in response to XAV939, which is consistent with previous observations (Fig. 1B).

To explore the XAV939-induced proteomic changes between 2D- and 3D-cultured SW480 cells, we calculated the iTRAQ ratios of 116/115 versus 114/113 (3D culture treated with XAV939/3D culture versus 2D culture treated with XAV939/2D culture), as shown in Table S4 in the Supplementary Information. The log_2_ fold-change distribution for iTRAQ ratios 116/115 versus 114/113 was tightly clustered around zero, indicating that the majority of proteins did not change in abundance (Fig. S1B in Supplementary Information). As a result, 24 significantly changed proteins were identified (fold-change >1.3 and p-value < 0.05), of which 10 proteins were up-regulated, and 14 proteins were down-regulated (red dots in Fig. 5B). The relative protein expression levels of these proteins are shown in Fig. 5C.

Table 2 provides detailed information about the identified proteins, including their GO terms. These proteins are involved in metabolic processes (DHTKD1, LDHA, ALDH1A3, and CYCS), protein transport (GOLGA7B, SEC24B, GOPC, and EEA1), DNA replication/transcription (HMGN2, ORC5, GTF2F1, and RBBP5), cell adhesion/cytoskeleton (CADM4, GSN, and DCHS2), apoptosis (TAX1BP1 and ANP32B), protein modification (PPME1 and UBLCP1), Wnt/β-catenin signaling (GSK3A/B), and other processes (HPGD, S100A6 and NUP50).

**Table 2:** Significantly changed proteins between 2D- and 3D-cultured SW480 cells upon treatment with XAV939.

Protein ID	Gene name	Protein name	GO terms			3D_XAV939/3D: 2D_XAV939/2D	
			Biological Process	Molecular Function		Log_2_ ratio	P-value
**Up**-**regulated proteins**							
P15428	HPGD	15-hydroxyprostaglandin dehydrogenase [NAD(+)]	Lipid metabolism		Oxidoreductase	0.75	0.014
Q96HY7	DHTKD1	Probable 2-oxoglutarate dehydrogenase E1 component DHKTD1	Metabolic process		Oxidoreductase	0.63	0.019
Q2TAP0	GOLGA7B	Golgin subfamily A member 7B	Protein transport		Transferase	0.49	0.035
O95487	SEC24B	Protein transport protein Sec24B	Protein transport		Protein/Metal ion binding	0.47	0.046
Q8NFZ8	CADM4	Cell adhesion molecule 4	Cell adhesion		Receptor binding	0.46	0.005
Q9Y570	PPME1	Protein phosphatase methylesterase 1	Protein modification		Hydrolase	0.46	0.021
P06396	GSN	Gelsolin	Cytoskeleton reorganization		Actin binding	0.39	0.001
Q86VP1	TAX1BP1	Tax1-binding protein 1	Apoptosis		Protein/Metal ion binding	0.39	0.012
Q8WVY7	UBLCP1	Ubiquitin-like domain-containing CTD phosphatase 1	Protein modification		Hydrolase	0.38	0.020
P06703	S100A6	Protein S100-A6	Signal transduction		Protein/Metal ion binding	0.38	0.011
**Down**-**regulated proteins**							
Q6V1P9	DCHS2	Protocadherin-23	Cell adhesion		Metal ion binding	−1.79	0.008
Q9HD26	GOPC	Golgi-associated PDZ and coiled-coil motif-containing protein	Protein transport		Protein binding	−1.16	0.018
P05204	HMGN2	Non-histone chromosomal protein HMG-17	DNA replicatoin		DNA binding	−0.90	0.050
Q9UKX7	NUP50	Nuclear pore complex protein Nup50	Intracellular transport		Ran GTPase binding	−0.74	0.038
P49840	GSK3A	Glycogen synthase kinase-3 alpha	Wnt signaling pathway		Protein kinase	−0.53	0.001
P49841	GSK3B	Glycogen synthase kinase-3 beta	Wnt signaling pathway		Protein kinase	−0.49	0.001
O43913	ORC5	Origin recognition complex subunit 5	DNA replicatoin		DNA binding	−0.44	0.001
P00338	LDHA	L-lactate dehydrogenase A chain	Metabolic process		Oxidoreductase	−0.43	0.008
P35269	GTF2F1	General transcription factor IIF subunit 1	DNA transcription		DNA binding	−0.40	0.029
Q15291	RBBP5	Retinoblastoma-binding protein 5	DNA transcription		DNA binding	−0.40	0.010
P47895	ALDH1A3	Aldehyde dehydrogenase family 1 member A3	Metabolic process		Oxidativereductase	−0.40	0.002
P99999	CYCS	Cytochrome c	Metabolic process		Elctron transport	−0.40	0.001
Q15075	EEA1	Early endosome antigen 1	Protein transport		Lipid binding	−0.38	0.028
Q92688	ANP32B	Acidic leucine-rich nuclear phosphoprotein 32 family member B	Chromatin remodeling/Apoptosis		Protein binding	−0.38	0.050

Notes: GO terms determined using PANTHER analysis tool. 3D_XAV939: 3D culture treated with XAV939, 3D:3D culture, 2D_XAV939: 2D culture treated with XAV939, 2D: 2D culture. Abbreviations: GO, Gene ontology.

**Figure 6:**
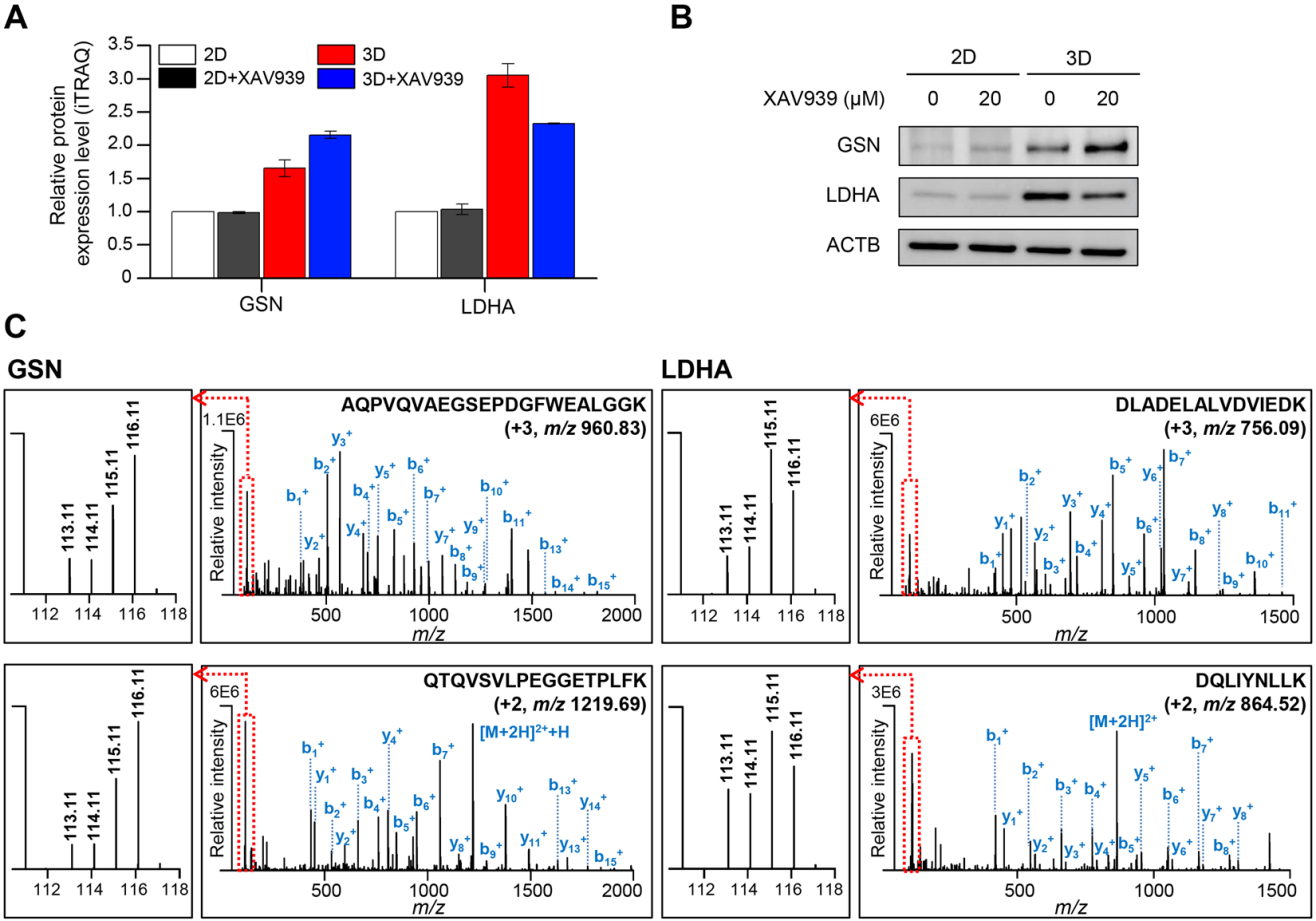
Validation of GSN and LDHA. (**A**) Relative protein expression levels of GSN and LDHA from iTRAQ data. Results are expressed as fold change, and protein expression levels were normalized to 2D culture. Values are shown as the means ± SEM. GSN, gelsolin; LDHA, lactate dehydrogenase A. (**B**) Validation of GSN and LDHA in 2D- and 3D-cultured SW480 cells either treated or untreated with XAV939 using western blot analysis. Full-length blots are presented in Supplementary Fig. S4. (**C**) Representative MS/MS spectra of AQPVQVAEGSEPDGFWEALGGK and QTQVSVLPEGGETPLFK for identified GSN and DLADELALVDVIEDK and DQLIYNLLK for identified LDHA. Left boxes represent spectra of iTRAQ reporter ions. 113.11, 2D culture; 114.11, 2D culture treated with XAV939; 115.11, 3D culture; 116.11, 3D culture treated with XAV939.

Of these 24 proteins, four proteins were previously proposed as TNKS protein interactors (GSK3A/B and TAX1BP1)^35,36^ or a Wnt/β-catenin signaling-target protein (HPGD) in 2D culture-based assays^37,38^, while the other proteins are novel XAV939-induced proteins. We are particularly interested in GSN and LDHA because XAV939-induced changes in their expression levels occurred only in 3D-cultured cells. We then verified the iTRAQ data of GSN and LDHA using western blot analysis (Fig. 6A,B). Representative MS/MS spectra for tryptic peptides of GSN and LDHA are shown in Fig. 6C.

GSN (gelsolin) is an actin-binding protein that regulates cytoskeleton dynamics and various cellular signalings, such as differentiation, proliferation, invasion, and apoptosis^39^. GSN has been shown to have dual roles as both a tumor promoter and suppressor, depending on the type of cancer cells^40–42^. Upon treatment of XAV939, we observed the up-regulation of GSN in 3D, but not in 2D culture (Fig. 6A, 2D, 1; 2D_XAV939, 0.99-fold; 3D, 1.66-fold; 3D_XAV939, 2.15-fold). This is interesting in the context of previous results showing that the overexpression of GSN inhibited the proliferation and invasion of renal cancer cells and promoted apoptosis in cardiac myocyte through the GSN/HIF-1α/DNase І pathway^42,43^. A possible explanation is that the up-regulation of GSN led to the growth inhibition of CRC cells in 3D culture upon treatment with XAV939. Also, the up-regulation of GSN was also observed in 3D compared with 2D culture, which was proposed in a previous report to be the result of the hypoxic conditions^44^.

LDHA belongs to the lactate dehydrogenase family and converts pyruvate to lactate in the final step of the glycolytic pathway^45^. The enhanced glycolytic activity has long been known as a hallmark of cancer because tumor cells rely on aerobic glycolysis for the production of energy, which is called the Warburg effect^45,46^. Therefore, the inhibition of LDHA suppresses tumor progression by alteration of energy metabolism and induction of oxidative stress^45^. We observed the up-regulation of glycolysis (Fig. 4A and Table 1) and LDHA levels (Fig. 3B) in 3D compared to 2D culture. These results indicate that the 3D culture system used in this study more closely represented the metabolic states of tumors *in vivo* than that of 2D culture. Interestingly, LDHA was significantly down-regulated in 3D-cultured cells in the presence of XAV939, whereas 2D-cultured cells showed no significant differences in LDHA expression (Fig. 6A, 2D, 1; 2D_XAV939, 1.03-fold; 3D, 3.05-fold; 3D_XAV939, 2.32-fold). These results suggest that XAV939 treatment induced the reduction of glycolysis via the down-regulation of LDHA in 3D culture, resulting in suppression of cell growth. Our results are consistent with a previous study in which a Wnt inhibition-mediated metabolic change to reduce glycolysis suppressed the growth of CRC cells in a xenograft tumor model^47^. In addition to LDHA, ALDH1A3 (aldehyde dehydrogenase 1 family member A3), which participates in the glycolytic pathway, also showed similar expression patterns in 2D- and 3D-cultured cells after treatment with XAV939 (Fig. 5C, 2D, 1; 2D_XAV939, 0.91-fold; 3D, 1.79-fold; 3D_XAV939, 1.23-fold).

Our quantitative profiling of XAV939-target proteins based on 2D and 3D cell culture could be useful for the discovery and development of new drugs for CRC. Nevertheless, it remains unclear why XAV939-target proteins are differentially expressed in 2D- and 3D-cultured cells. One conceivable mechanism is functional changes in β-catenin signaling that depend on the cellular microenvironments, hypoxia, and normoxia conditions^48^. In normoxia, β-catenin directly interacts with TCF-4 (T-cell factor-4) and enhances TCF-4–mediated transcription. In hypoxic conditions, however, the interaction of β-catenin and HIF-1 is predominant over the β-catenin-TCF-4 complex. Therefore, the HIF-1-dependent transcription is improved, but the TCF-4 transcriptional activity is reduced. Hypoxia conditions are one notable characteristic of 3D culture, while 2D-cultured cells are commonly grown under normoxic conditions. It is therefore reasonable to argue that the expression of TCF-4/HIF-1-target genes could be regulated differentially in 2D versus 3D culture conditions when β-catenin protein levels are decreased by XAV939. The fact that LDHA is one of the HIF-1-target genes supports this suggestion^46^. Further investigations are required to fully understand these mechanisms.

## Conclusion

Herein, we presented a comparative proteomic analysis of 2D- and 3D-cultured SW480 cells with a focus on the XAV939-induced changes. We observed the up-regulation of glycolytic proteins and down-regulation of proteins associated with cell growth in 3D-cultured SW480 cells compared to 2D-cultured SW480 cells. This supports the fact that 3D cell culture models more appropriately represent cancer phenotypes *in vivo* than 2D cell culture models. Compared to previous proteomic studies of 3D-cultured cells, it is noticeable that mitochondrial oxidative phosphorylation was either activated or inactivated depending on the 3D cell culture models. This was possibly affected by the type of cells, 3D cell culture systems, spheroid sizes, or other factors. In addition, we showed that XAV939 suppresses the growth of SW480 cells in 3D culture, but not in 2D culture, even though Wnt/β-catenin signaling was successfully impaired by XAV939 in both 2D- and 3D-cultured SW480 cells. We identified the novel XAV939-responsive proteins involved in the growth inhibitory effects of XAV939 in 3D-cultured SW480 cells including gelsolin and lactate dehydrogenase A. These results provide novel insight into the mechanisms of XAV939-induced growth inhibition of 3D-cultured SW480 cells.

## Materials and Methods

### Materials and Chemicals

Ammonium bicarbonate (NH_4_HCO_3_, ABC), dithiothreitol (DTT), iodoacetamide (IAA), L-cysteine, and formic acid were purchased from Sigma (St. Louis, MO, USA). Sequencing-grade trypsin was obtained from Promega Corp. (Madison, WI, USA). HPLC-grade acetonitrile (ACN) and water (Burdick & Jackson, Ulsan, Korea) were used for a binary nLC separation of tryptic digests. For the online 2D-nLC-MS/MS run, fused-silica capillaries (25, 50, 75, and 100-μm I.D., 360-μm O.D.) for the LC column and tubing connections were obtained from Technology LLC (Phoenix, AZ, USA). Six-port switching valves, fittings, adapters, and PEEK tubing were purchased from Upchurch Scientific® (Oak Harbor, WA, USA) or IDEX Health & Science LCC.

### Cell Culture and Drug Treatment

The CRC cell line SW480 was purchased from the American Type Culture Collection (ATCC, Manassas, VA). The cells were cultured in RPMI1640 with 10% fetal bovine serum and 1% penicillin/streptomycin. The same media were used in 2D and 3D culture. For the 2D culture, 1 × 10^6^ cells were seeded in a 100-mm culture dish and treated with either 20 μM of XAV939 (Sigma Aldrich, X3004) or DMSO for 72 h. For the 3D culture, soft agar colony formation kits (Cell Biolabs, Inc.) were used according to the modified manufacturer’s instructions. Cells were embedded in 0.4% soft agar at a concentration of 2 × 10^6^ cells in a 6-well plate and treated with either 20 μM of XAV939 or DMSO for 6 days.

### Cytotoxicity Assay

The cell viability in 2D culture was assessed by Cell Counting Kit-8 (Dojindo Molecular Technologies, Inc.) according to the manufacturer’s instructions. Briefly, SW480 cells were cultured with various concentrations of XAV939 for 72 h. Subsequently, 10 μL of CCK8 reagent was incubated with cells for 3 h, and then the absorbance at 450 nm was measured using a Spectramax® i3 (Molecular Devices). To determine the cell viability in 3D culture, a CytoSelect™ 96-Well Cell Transformation Assay (Cell Biolabs, Inc.) was performed according to the manufacturer’s instructions. Briefly, SW480 cells were embedded in 0.4% soft agar and treated with various concentrations of XAV939 for 6 days. The cells were then lysed and incubated with CyQuant GR dye. The fluorescence is proportional to the number of cells and was read using a Spectramax® i3 (Molecular Devices) with a 485/520-nm filter.

### Cell Lysis and Protein Extraction

The cell pellets were resuspended with cell lysis buffer containing 8 M UREA, 50 mM Tris-HCl (pH 8.0), 75 mM NaCl, and a cocktail of protease inhibitors. The 2D- and 3D-cultured cells were sonicated (2-sec pulses with 3-sec rest) and then centrifuged for 15 min at 12000 rpm to remove cell debris. The supernatants were collected, and the protein concentrations were determined using a bicinchoninic acid (BCA) assay. The extracted proteins were stored at −80 °C until further analysis, including the proteomic analysis or Western blotting.

### Western Blot

Equal amounts of protein from each sample were separated by 4–20% sodium dodecyl sulfate-polyacrylamide gel electrophoresis (SDS-PAGE), transferred to nitrocellulose membranes, and incubated with the following primary antibodies: anti-axin2 (Cell Signaling, #2151), anti-β-catenin (Cell Signaling, #8480), anti-lactate dehydrogenase A (Cell Signaling, #2012), anti- phosphoglycerate kinase 1 (Santa Cruz, sc-130335), anti-glyceraldehyde 3-phosphate dehydrogenase (Santa Cruz, sc-25778), anti-nucleophosmin (Abcam, ab10530), anti-nucleolin (Cell Signaling, 14574), anti-drebrin 1 (Abnova, H00001627-M03), anti-glycogen synthase kinase 3 α/β (Cell Signaling, #5676), and anti-gelsolin (Cell Signaling, #12953). Anti-β-actin (Sigma-Aldrich, A5441) was used as a loading control. After washing, the blots were labeled with horseradish peroxidase-conjugated secondary antibodies and visualized with the ECL reagent (GE Healthcare) according to the manufacturer’s protocol.

### Proteolytic Digestion and iTRAQ Labeling

A 50-μg amount of proteins from each sample was used for proteomic analysis. Proteins were reduced with 10 mM dithiothreitol (DTT) for 2 h at 37 °C and alkylated with 20 mM iodoacetamide (IAA) for 30 min at room temperature in the dark. The remaining IAA was removed by the addition of excess L-cysteine. To reduce the final concentration of urea to 1 M, the mixtures were diluted with 50 mM ammonium bicarbonate buffer. Proteins were digested with trypsin (1:50, w/w) for 18 h at 37 °C, and then 1% formic acid (v/v) was added to stop the digestion. The tryptic peptides were desalted on 1-mg OASIS HLB cartridges and completely dried in a SpeedVac (Concentrator, Eppendorf).

For iTRAQ analysis, the dried peptides were dissolved in 30 μl of 0.5 M triethylammonium bicarbonate (TEAB) buffer. The iTRAQ reagents were used to label individual peptide samples as follows: 2D-cultured cells, 113 tag; 2D-cultured cells treated with 20 μM XAV939, 114 tag; 3D-cultured cells, 115 tag; 3D-cultured cells treated with 20 μM XAV939, 116 tag. After incubation for 2 h at room temperature, the reaction was stopped by addition of 1% formic acid (v/v). The four iTRAQ-labeled samples were combined in one tube and desalted on 10-mg OASIS HLB cartridges. The resulting peptides were dried in a SpeedVac and reconstituted with 0.1% formic acid (FA) in water for online 2D-nLC-MS/MS analysis. Two independent biological replicates were performed, and each sample was analyzed in duplicate online 2D-nLC-MS/MS runs.

### Online 2D-nLC-MS/MS

Nanoflow liquid chromatography-electrospray-tandem mass spectrometry (nLC-MS/MS) was performed using a Q-Exactive FT orbitrap-mass spectrometer (Thermo Fisher Scientific) coupled to a capillary LC system (Agilent Technologies 1260). For online 2D-nLC operation, an analytical column and biphasic trap columns were prepared in-house, as previously described^49^. The reverse-phase (RP) analytical column (150 mm in length, 75 μm I.D.) was packed with C18 resin (3-μm particle size, 100-Å pore size). The biphasic trap columns (40 mm in length, 200-μm I.D.) were packed with 50 mm of C18 resin (5-μm particle size, 200 Å pore size) and then 150 mm of strong cation exchange (SCX) resin (5-μm particle size, 200-Å pore size).

The peptide samples were loaded onto the SCX resins of the biphasic trap column and eluted with 12-step salt gradients (0, 15, 20, 22.5, 25, 27.5, 30, 40, 50, 100, 200 mM, and 1 M ammonium bicarbonate buffer containing 0.1% FA). After each salt fractionation, eluted peptides were bounded on RP resins, followed directly by RP gradient elution with mobile phase A (0.1% FA in water) and mobile phase B (2% water and 0.1% FA in acetonitrile). The RPLC separation was performed at a column flow rate of 200 nL/min with the following gradient conditions: 2% B (0–10 min), 2–10% B (10–11 min), 10–17% B (10–15 min), 17–33% B (15–85 min), 33–90% B (15–88 min), 90% B (88–103 min), 90–2% B (103–105 min), 2% B (105–120 min).

Electrospray ionization (ESI) was performed at a spray voltage of 2.5 kV. MS precursor ions were analyzed in positive mode with a scan range of 300 to 1800 *m/z*. MS spectra were acquired with a resolution of 70,000, AGC target of 3 × 10^6^, and maximum injection time of 80 ms. The twelve most intense precursor ions were selected for data-dependent MS/MS scans with exclusions of singly charged ions. Selected precursor ions were fragmented by high-energy collision dissociation (HCD) with NCE 27. For the MS/MS scans, the first fixed mass was set at 100 *m/z*, and the resolution was 35,000. Dynamic exclusion was allowed for 30 s.

### Data Analysis and Bioinformatics Analysis

The acquired raw data were analyzed using the MaxQuant search engine 1.6.0.1 for protein identification and iTRAQ quantification. The data were searched against the UniProt human database (last modified on April 22, 2017). The search parameters were set with the following conditions: two missed cleavage sites for trypsin, precursor ion mass tolerance of 20 ppm, fragment ion mass tolerance of 20 ppm, carbamidomethylation of cysteine (+57.021 Da), and iTRAQ modification of N-terminal residue (+304.205 Da) as fixed modifications, oxidation of methionine (+15.995 Da), carbamylation of lysine (+43.006 Da), acetylation of N-terminal residue (+42.011 Da), and iTRAQ modification of lysine (+304.205 Da) as variable modifications.

Proteins were identified with at least two unique peptides per protein. The false discovery rate (FDR) of peptides and proteins was set to 0.01. Data processing and statistical analysis were performed using the software Perseus 1.5.8.5. Reverse and contaminant proteins were excluded, and iTRAQ ratios were log_2_ transformed. The dataset was normalized by subtracting the median, and missing values were imputed using a normal distribution. We used a fold-change threshold of 1.6 or 1.3 to determine differently expressed proteins.

All datasets were subjected to a student’s t-test, and p-values less than 0.05 were considered as statistically significant. The functional enrichment analysis of significantly changed proteins was performed using GO analysis (http://www.geneontology.org/) and the PANTHER analysis tool. A KEGG pathway analysis was applied, followed by the Search Tool for the Retrieval of Interacting Genes/Proteins (STRING).

## Electronic supplementary material

Supplementary Information

Supplementary Dataset

## References

[1] Edmondson R, Broglie JJ, Adcock AF, Yang L (2014). Three-dimensional cell culture systems and their applications in drug discovery and cell-based biosensors. Assay Drug Dev Technol..

[2] Lai Y, Asthana A, Kisaalita W (2011). Biomarkers for simplifying HTS 3D cell culture platforms for drug discovery: the case for cytokines. Drug Discov Today.

[3] Antoni D, Burckel H, Josset E, Noel G (2015). Three-dimensional cell culture: a breakthrough *in vivo*. Int J Mol Sci..

[4] Justice B, Badr N, Felder R (2009). 3D cell culture opens new dimensions in cell-based assays. Drug Discov Today.

[5] Nath S, Devi G (2016). Three-dimensional culture systems in cancer research: Focus on tumor spheroid model. Pharmacol Ther..

[6] Rimann M, Graf-Hausner U (2012). Synthetic 3D multicellular systems for drug development. Curr Opin Biotechnol..

[7] Pruksakorn D (2010). Metabolic alteration of HepG2 in scaffold-based 3-D culture: proteomic approach. Proteomics.

[8] Wu Y, Tang J, Zhao P, Chen Z, Jiang J (2009). Morphological changes and molecular expressions of hepatocellular carcinoma cells in three-dimensional culture model. Exp Mol Pathol..

[9] Lovitt C, Shelper T, Avery V (2014). Advanced cell culture techniques for cancer drug discovery. Biology.

[10] Kislinger T, Emili A (2005). Multidimensional protein identification technology: current status and future prospects. Expert Rev Proteomics..

[11] Wolters D, Washburn M, Yates J (2001). An automated multidimensional protein identification technology for shotgun proteomics. Anal Chem..

[12] Rauniyar N, Yates J (2014). Isobaric labeling-based relative quantification in shotgun proteomics. J Proteome Res..

[13] Chahrour O, Cobice D, Malone J (2015). Stable isotope labelling methods in mass spectrometry-based quantitative proteomics. J Pharm Biomed Anal..

[14] Morrison B (2012). Proteomic comparison of MCF-7 tumoursphere and monolayer cultures. PLoS One.

[15] He W (2014). Proteomic comparison of 3D and 2D glioma models reveals increased HLA-E expression in 3D models is associated with resistance to NK cell-mediated cytotoxicity. J Proteome Res..

[16] Yue X, Lukowski JK, Weaver EM, Skube SB, Hummon AB (2016). Quantitative Proteomic and Phosphoproteomic Comparison of 2D and 3D Colon Cancer Cell Culture Models. J Proteome Res..

[17] Hongisto V (2013). High-throughput 3D screening reveals differences in drug sensitivities between culture models of JIMT1 breast cancer cells. PLoS One.

[18] Longati P (2013). 3D pancreatic carcinoma spheroids induce a matrix-rich, chemoresistant phenotype offering a better model for drug testing. BMC Cancer.

[19] Karlsson H, Fryknäs M, Larsson R, Nygren P (2012). Loss of cancer drug activity in colon cancer HCT-116 cells during spheroid formation in a new 3-D spheroid cell culture system. Exp Cell Res..

[20] Loessner D (2010). Bioengineered 3D platform to explore cell-ECM interactions and drug resistance of epithelial ovarian cancer cells. Biomaterials.

[21] Imamura Y (2015). Comparison of 2D- and 3D-culture models as drug-testing platforms in breast cancer. Oncol Rep..

[22] Trédan O, Galmarini C, Patel K, Tannock I (2007). Drug resistance and the solid tumor microenvironment. J Natl Cancer Inst..

[23] Senkowski W (2015). Three-Dimensional Cell Culture-Based Screening Identifies the Anthelmintic Drug Nitazoxanide as a Candidate for Treatment of Colorectal Cancer. Mol Cancer Ther..

[24] Fayad W (2011). Identification of agents that induce apoptosis of multicellular tumour spheroids: enrichment for mitotic inhibitors with hydrophobic properties. Chem Biol Drug Des..

[25] Adcock A, Trivedi G, Edmondson R, Spearman C, Yang L (2015). Three-Dimensional (3D) Cell Cultures in Cell-based Assays for *in-vitro* Evaluation of Anticancer Drugs. J Anal Bioanal Tech..

[26] Polakis P (2007). The many ways of Wnt in cancer. Curr Opin Genet Dev..

[27] Barker N, Clevers H (2006). Mining the Wnt pathway for cancer therapeutics. Nat Rev Drug Discov..

[28] Powell S (1992). APC mutations occur early during colorectal tumorigenesis. Nature.

[29] Huang SM (2009). Tankyrase inhibition stabilizes axin and antagonizes Wnt signalling. Nature.

[30] Ferri M (2017). Targeting Wnt-driven cancers: Discovery of novel tankyrase inhibitors. Eur J Med Chem..

[31] Krishnamurthy N, Kurzrock R (2018). Targeting the Wnt/beta-catenin pathway in cancer: Update on effectors and inhibitors. Cancer Treat Rev..

[32] Wu X, Luo F, Li J, Zhong X, Liu K (2016). Tankyrase 1 inhibitior XAV939 increases chemosensitivity in colon cancer cell lines via inhibition of the Wnt signaling pathway. Int J Oncol..

[33] Bao R (2012). Inhibition of tankyrases induces Axin stabilization and blocks Wnt signalling in breast cancer cells. PLoS One.

[34] Waaler J (2012). A novel tankyrase inhibitor decreases canonical Wnt signaling in colon carcinoma cells and reduces tumor growth in conditional APC mutant mice. Cancer Res..

[35] Sbodio J, Chi N (2002). Identification of a tankyrase-binding motif shared by IRAP, TAB182, and human TRF1 but not mouse TRF1. NuMA contains this RXXPDG motif and is a novel tankyrase partner. J Biol Chem..

[36] Li X (2017). Proteomic Analysis of the Human Tankyrase Protein Interaction Network Reveals Its Role in Pexophagy. Cell Rep..

[37] Smartt H (2012). β-catenin represses expression of the tumour suppressor 15-prostaglandin dehydrogenase in the normal intestinal epithelium and colorectal tumour cells. Gut.

[38] Mehdawi L, Prasad C, Ehrnström R, Andersson T, Sjölander A (2016). Non-canonical WNT5A signaling up-regulates the expression of the tumor suppressor 15-PGDH and induces differentiation of colon cancer cells. Mol Oncol..

[39] Li G, Arora P, Chen Y, McCulloch C, Liu P (2012). Multifunctional roles of gelsolin in health and diseases. Med Res Rev..

[40] Abedini M (2014). Cell fate regulation by gelsolin in human gynecologic cancers. Proc Natl Acad Sci USA.

[41] Deng R (2015). Gelsolin regulates proliferation, apoptosis, migration and invasion in human oral carcinoma cells. Oncol Lett..

[42] Zhu X, Cai L, Meng Q, Jin X (2015). Gelsolin inhibits the proliferation and invasion of the 786-0 clear cell renal cell carcinoma cell line *in vitro*. Mol Med Rep..

[43] Li GH (2009). Gelsolin regulates cardiac remodeling after myocardial infarction through DNase I-mediated apoptosis. Circ Res..

[44] Yeh Y (2016). Hypoxia Augments Increased HIF-1α and Reduced Survival Protein p-Akt in Gelsolin (GSN)-Dependent Cardiomyoblast Cell Apoptosis. Cell Biochem Biophys..

[45] Le A (2010). Inhibition of lactate dehydrogenase A induces oxidative stress and inhibits tumor progression. Proc Natl Acad Sci USA.

[46] Miao P, Sheng S, Sun X, Liu J, Huang G (2013). Lactate dehydrogenase A in cancer: a promising target for diagnosis and therapy. IUBMB Life.

[47] Pate KT (2014). Wnt signaling directs a metabolic program of glycolysis and angiogenesis in colon cancer. EMBO J..

[48] Kaidi A, Williams A, Paraskeva C (2007). Interaction between beta-catenin and HIF-1 promotes cellular adaptation to hypoxia. Nat Cell Biol.

[49] Kang D, Nam H, Kim Y, Moon M (2005). Dual-purpose sample trap for on-line strong cation-exchange chromatography/reversed-phase liquid chromatography/tandem mass spectrometry for shotgun proteomics. Application to the human Jurkat T-cell proteome. J Chromatogr A.

