# Supplementary material for: Quantitative Proteomic Analysis of 2D and 3D Cultured Colorectal Cancer Cells: Profiling of Tankyrase Inhibitor XAV939-Induced Proteome
Source: Sci Rep. 2018 Sep 5;8:13255. doi: 10.1038/s41598-018-31564-6 (PMC6125324; doi:10.1038/s41598-018-31564-6)
Supplement: Supplementary file 1 — Supplementary Information [file 41598_2018_31564_MOESM1_ESM.docx]

**Supporting Information**

Quantitative Proteomic Analysis of 2D and 3D Cultured Colorectal Cancer Cells: Profiling of Tankyrase Inhibitor XAV939-Induced Proteome

Young Eun Kim,^1,§^ Hyo Jin Jeon,^2, 3,§^ Dahee Kim,^2^ Sun Young Lee,^1,4^ Ki Young Kim,^2^ Jongki Hong,^4^ Pil Jae Maeng,^3^ Kwang-Rok Kim,^2,*^ Dukjin Kang^1,*^

^1^Center for Bioanalysis, Division of Chemical and Medical Metrology, Korea Research Institute of Standards and Science, Daejeon, 34113, Korea

^2^Therapeutic & Biotechnology Division, Korea Research Institute of Chemical Technology, Daejeon, 34114, Korea

^3^Department of Microbiology and Molecular Biology, Chungnam National University, Daejeon, 34134, Korea

^4^College of Pharmacy, Kyung Hee University, Seoul, 02447, Korea

^§^Both authors contributed equally to this study

**Supporting Figure**

**Figure S1.** Histograms of Log_2_-fold change for (A) 3D culture/2D culture and (B) 3D culture treated with XAV939/3D culture verse 2D culture treated with XAV939/2D

**Figure S2.** Full-length blots corresponding to Fig.1B in the main text.

**Figure S3.** Full-length blots corresponding to Fig.3B in the main text.

**Figure S4.** Full-length blots corresponding to Fig.6B in the main text.

**Supporting Tables**

All tables listed here are shown in a supporting Excel file.

**Table S1.** List of all quantified proteins with Log_2_ (3D culture/2D culture) ratio and p-value

**Table S2.** List of significantly up-regulated proteins in 3D culture compared with 2D culture

**Table S3.** List of significantly down-regulated proteins in 3D culture compared with 2D culture

**Table S4.** List of all quantified proteins with Log_2_ (3D_XAV939/3D:2D_XAV939/2D) ratio and p-value

**Supporting Figure S1**

**
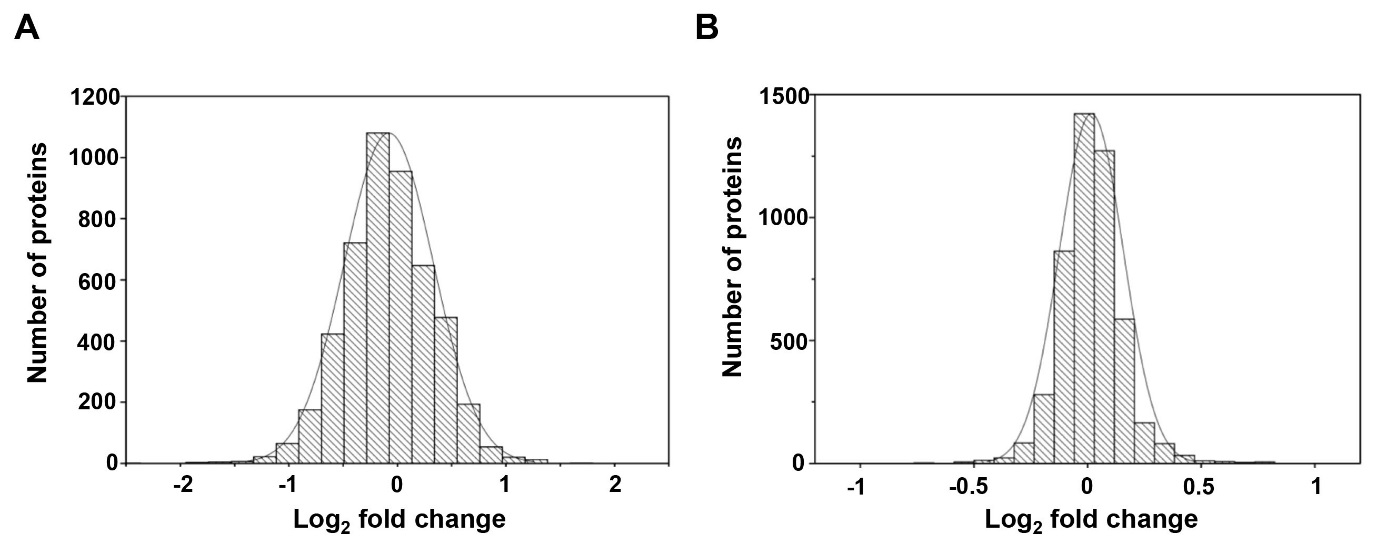
**

**Figure S1.** Histograms of Log_2_-fold change for **(A)** 3D culture/2D culture and **(B)** 3D culture treated with XAV939/3D culture verse 2D culture treated with XAV939/2D

**Supporting Figure S2**

**Figure S2.** Full-length blots corresponding to Fig.1B in the main text.

**Supporting Figure S3**

**Figure S3.** Full-length blots corresponding to Fig.3B in the main text.

**Supporting Figure S4**

**Figure S4.** Full-length blots corresponding to Fig.6B in the main text.
